# Proinflammatory plasticity towards Th17 paradigm of regulatory T cells consistent with elevated prevalence of *TGFBR2* variants in elderly patients with primary immune thrombocytopenia

**DOI:** 10.1186/s12865-023-00541-8

**Published:** 2023-04-07

**Authors:** Jingjing Cao, Yanxia Zhan, Lili Ji, Pu Chen, Luya Cheng, Feng Li, Xibing Zhuang, Zhihui Min, Lihua Sun, Fanli Hua, Hao Chen, Boting Wu, Yunfeng Cheng

**Affiliations:** 1 Department of Hematology, Zhongshan Hospital, Fudan University, Shanghai, 200032 China; 2 Department of Laboratory Medicine, Zhongshan Hospital, Fudan University, Shanghai, 200032 China; 3 Department of Hematology, Zhongshan Hospital Qingpu Branch, Fudan University, Shanghai, 201700 China; 4 Center for Tumor Diagnosis and Therapy, Jinshan Hospital, Fudan University, Shanghai, 201508 China; 5 Institute of Clinical Science, Zhongshan Hospital, Fudan University, Shanghai, 200032 China; 6 Department of Thoracic Surgery, Zhongshan Hospital Xuhui Branch, Fudan University, Shanghai, 200031 China; 7 Department of Transfusion, Zhongshan Hospital, Fudan University, Shanghai, 200032 China

**Keywords:** Primary immune thrombocytopenia, Regulatory T cells, Proinflammatory plasticity, *TGFBR2* variants

## Abstract

**Background:**

Primary immune thrombocytopenia (ITP) is characterized for the skewed Th differentiation towards Th1 and Th17 cells as well as the impaired number and function of regulatory T cells (Tregs). Tregs are capable of co-expressing effector Th markers in different inflammatory milieu, which probably indicates Treg dysfunction and incompetence to counter over-activated immune responses.

**Methods:**

Ninety-two primary ITP patients from March 2013 to December 2018 were included, and proinflammatory plasticity in different Treg compartments, age groups, and *TGFBR2* variant carrier status were investigated.

**Results:**

Patients were categorized into elderly (n = 44) and younger (n = 48) groups according to an age of 50 years at disease onset. The overall remission rate was 82.6% after first-line regimens, including 47.8% complete remission. *TGFBR2* variants were found in 7 (7.6%) patients with three V216I and four T340M heterozygote carriers. ITP patients demonstrated elevated co-expression of IL-17 and decreased co-expression of both IFN-γ and IL-13 than health control (all *p* < 0.01). The elderly group demonstrated elevated prevalence of *TGFBR2* variants (*p* = 0.037) and elevated co-expression of IL-17 (*p* = 0.017) in Tregs, while female predominance was found in the younger group (*p* = 0.037). In the elderly group, *TGFBR2* variant carriers demonstrated further elevated co-expression of IL-17 (*p* = 0.023) and decreased co-expression of both IFN-γ (*p* = 0.039) and IL-13 (*p* = 0.046) in the aTreg compartment.

**Conclusions:**

Our findings revealed additional aberrations of Treg proinflammatory plasticity in elderly primary ITP patients, and highlighted the potential role of Treg dysfunction and senescence in the pathogenesis and management among these patients.

## Background

As the most prevalent acquired bleeding disorder in adults, primary immune thrombocytopenia (ITP) displays two peaks of incidence, one between 20 and 30 years of age with a slight female predominance, the other after 60 with equal sex distribution [1, 2]. Due to elevated bleeding risk and limited treatment options, primary ITP in the elderly is often considered to be a clinical challenge [3, 4]. Recently, immune senescence and its biological impact upon diseases have been caught into academic limelight [5]. However, the aberration in T cell biology among elderly primary ITP patients is yet to be elaborated.

In addition to direct platelet injuries inflicted by antiplatelet antibodies and cytotoxic T cells, the abnormalities in T helper (Th) cells, including the skewed Th differentiation towards Th1 and Th17 cells as well as the impaired number and function of regulatory T cells (Tregs), are the keystones in the pathogenesis of primary ITP [6–9]. Tregs play vital roles in the maintenance of immune homeostasis, the inhibition of immune responses, and the establishment of self-tolerance [10–13]. In different inflammatory milieu, Tregs are capable of co-expressing effector Th markers including interferon-γ (IFN-γ), interleukin-13 (IL-13), and interleukin-17 (IL-17) [12, 14, 15]. This proinflammatory plasticity of Tregs is not only determined by specific inflammatory microenvironment of diseases [16], but also associated with genetic predisposition in essential signaling pathway molecules such as TGF-β receptors [17].

The present study investigated the proinflammatory plasticity in different Treg compartments among elderly primary ITP patients, and further evaluated the impact of *TGFBR2* variants on Treg differentiation and plasticity, thus intending to provide novel perspectives in the pathogenesis and management of primary ITP.

## Methods

### Study population

Primary ITP patients diagnosed in our institution from March 2013 to December 2018 were included in the present study. Primary ITP was defined as blood platelet count lower than 100 × 10^9^/L in the absence of other causes or disorders that may be associated with thrombocytopenia according to the International Working Group [18]. The exclusion criteria, in specific, were systemic connective tissue diseases, active infection, malignancies, and pregnancy. Clinical data were extracted from medical records. Primary ITP patients were categorized into younger (18–49 years) and elderly (50 years and over) groups according to their age at disease onset [19]. The treatment response to first-line regimens was categorized into complete remission (CR), partial remission (PR), and no remission (NR) based on the criteria of complete response, response, and no response by an International Working Group [18].

The present study was approved by the institutional Ethics Committee. Written informed consent was obtained from all participants conforming to the Declaration of Helsinki. Age- and sex-matched healthy volunteers were included as control group.

### Genotyping

Genomic DNA extracted from peripheral whole blood samples was amplified by routine polymerase chain reaction (PCR) procedure, and the PCR product was purified and sequenced by ABI 3730XL DNA Analyzer (Applied Biosystems, Waltham MA, USA) to determine the carrier status of 2 *TGFBR2* variants, p.Val216Ile/c.646G>A (rs56105708) and p.Thr340Met/c.1019C>T (rs34833812). Primer sequences for PCR are listed in Table 1.

**Table 1:** Primers for genotyping of *TGFBR2* variants

Genetic variant	Location	SNP	Forward primer	Reverse primer	PCR product
*TGFBR2* p.Val216Ile/c.646G>A	3p24.1	rs56105708	CATGAACCCACTTCCTGACA	CAGCAGCTCTGTGTTGTGGT	345 bp
*TGFBR2* p.Thr340Met/c.1019C>T	3p24.1	rs34833812	GCCAACAACATCAACC	CGTTCTTCACGAGGATA	475 bp

### Flow cytometry analysis

Peripheral blood mononuclear cells (PBMCs) were isolated from EDTA-anticoagulated whole blood samples by density-gradient centrifugation over Ficoll Hypaque gradients. Fixable Viability Stain 780 (BD Biosciences, Cat #565388) was used for the exclusion of dead cells. For the identification of Treg and Treg compartments, 1 × 10^6^ PBMCs were stained with surface CD4 FITC (BD Biosciences, Cat #555346), CD25 PE (BD Biosciences, Cat #560989), CD45RA BV480 (BD Biosciences, Cat #566155), and intracellular Foxp3 AF647 (BD Biosciences, Cat #560045). Tregs were defined as CD4^+^CD25^hi^Foxp3^+^ cells. Treg compartments, namely aTreg, rTreg, and nsTreg, were further defined as Foxp3^hi^CD45RA^−^, Foxp3^int^CD45RA^+^, and Foxp3^int^CD45RA^−^ cells, respectively among Tregs [20].

For the assessment of proinflammatory plasticity of Tregs, PBMCs were cultured in RPMI1640 medium supplemented with 10% heat-inactivated fetal bovine serum, 200U/ml penicillin, and 100 μg/ml streptomycin. After 5-h stimulation with Cell Stimulation Cocktail (eBioscience, Cat #00-4975-93), PBMCs were stained intracellularly with either IFN-γ PE/Dazzle 594 (BioLegend, Cat #502546), IL-13 PE-CY7 (BioLegend, Cat #501914), or IL-17 BV421 (BioLegend, Cat #512322) in addition to the staining protocol previously described for Treg compartments.

Flow cytometry analyses were performed on FACSAria III platform (BD Biosciences, Franklin Lakes NJ, USA). All data analyses were performed with FlowJo sofeware version 10.4 (FlowJo LLC, Ashland OR, USA).

### Statistical analysis

Continuous variables were evaluated for normal distribution using Shapiro–Wilk test and reported as mean ± SD or medians (interquartile ranges), as appropriate. Categorical variables were presented as frequencies (percentages). Differences between 2 groups were assessed by Student’s t test for normally distributed continuous data, Mann–Whitney U-test for non-normally distributed continuous data, and chi-square test or Fisher’s exact test for categorical data. Statistical significance was defined as 2-sided *p* < 0.05. Analysis was performed with SPSS Statistics 23 (IBM, NY, USA).

## Results

### Patient characteristics

From March 2013 to December 2018, 92 primary ITP patients with an onset age of 48 ± 19 years and platelet count of (14 ± 7) × 10^9^/L were included in the present study. The overall remission rate was 82.6% after first-line regimens, including 47.8% CR and 34.8% PR. Anti-nuclear antibody was detected in 25 (27.2%) patients, including 19 with a 1:100 titer and 6 with a 1:320 titer. Common *TGFBR2* variants were found in 7 (7.6%) patients, including three V216I and four T340M heterozygote carriers.

Lymphocyte subgroups, including CD19^+^, CD3^+^CD4^+^, CD3^+^CD8^+^, CD16^+^CD56^+^ compartment, were comparable between primary ITP patients and health control. Although total Tregs [(1.2 ± 0.9)% vs. (1.1 ± 0.3)%, *p* = 0.697] were comparable between primary ITP patients and health control, primary ITP patients demonstrated markedly elevated co-expression of IL-17 [(12.9 ± 9.4)% vs. (3.5 ± 1.4)%, *p* = 0.005] and decreased co-expression of both IFN-γ [(8.2 ± 5.0)% vs. (23.2 ± 5.5)%, *p* < 0.001] and IL-13 [(7.4 ± 4.6)% vs. (11.8 ± 4.7)%, *p* = 0.007] (Table 2).

**Table 2:** Demographic characteristics and Treg plasticity of primary ITP patients

	All ITP (n = 92)	Health control (n = 12)	*p* value	Onset 18–49 years (n = 48)	Onset over 50 years (n = 44)	*p* value
Female sex, n (%)	64 (69.6)	8 (66.7)	0.838	38 (79.2)	26 (59.1)	0.037
Age, years	48 ± 19	42 ± 14	0.272	33 ± 8	65 ± 10	< 0.001
Platelet count, × 10^9^/L	14 ± 7	205 ± 42	< 0.001	12 ± 8	16 ± 8	0.359
Response, n (%)						
CR	44 (47.8)	NA	NA	26 (54.2)	18 (40.9)	0.206
PR	32 (34.8)	NA	NA	15 (31.3)	17 (38.6)	0.457
NR	16 (17.4)	NA	NA	7 (14.6)	9 (20.5)	0.458
*TGFBR2* variants, n (%)	7 (7.6)	NA	NA	1 (2.1)	6 (13.6)	0.037
Positive ANA, n (%)	25 (27.2)	NA	NA	13 (27.1)	12 (27.3)	0.984
Lymphocyte subgroups, %						
CD19^+^	19 ± 8	19 ± 5	0.962	21 ± 6	16 ± 10	0.053
CD3^+^CD4^+^	36 ± 9	39 ± 6	0.418	35 ± 8	37 ± 11	0.389
CD3^+^CD8^+^	27 ± 10	23 ± 5	0.175	26 ± 4	27 ± 14	0.672
CD16^+^CD56^+^	13 ± 7	11 ± 3	0.143	12 ± 7	14 ± 7	0.591
Tregs, %						
CD4^+^CD25^hi^Foxp3^+^	1.2 ± 0.9	1.1 ± 0.3	0.697	1.1 ± 0.9	1.2 ± 0.9	0.792
CD4^+^CD25^hi^Foxp3^+^IFN-γ^+^	8.2 ± 5.0	23.2 ± 5.5	< 0.001	7.3 ± 5.4	8.3 ± 4.8	0.605
CD4^+^CD25^hi^Foxp3^+^IL-13^+^	7.4 ± 4.6	11.8 ± 4.7	0.007	7.3 ± 5.1	7.4 ± 4.5	0.966
CD4^+^CD25^hi^Foxp3^+^IL-17^+^	12.9 ± 9.4	3.5 ± 1.4	0.005	5.2 ± 3.0	16.9 ± 13.8	0.017

CR: complete remission; PR: partial remission; NR: no remission; ANA: anti-nuclear antibody; Treg: regulatory T cell

### Comparison between elderly and younger primary ITP patients

Female predominance was found in younger primary ITP patients with an onset age of 18–49 years (79.2% vs. 59.1%, *p* = 0.037). Comparing to younger patients, elderly primary ITP patients demonstrated elevated prevalence of *TGFBR2* variants (13.6% vs. 2.1%, *p* = 0.037), slightly decreased CD19^+^ B cell population [(16 ± 10)% vs. (21 ± 6)%, *p* = 0.053], and further elevated co-expression of IL-17 [(16.9 ± 13.8)% vs. (5.2 ± 3.0)%, *p* = 0.017] in Tregs (Table 2).

### Increased frequency of immunosuppressive cells in ITP patients with mutation

**Fig. 1:**
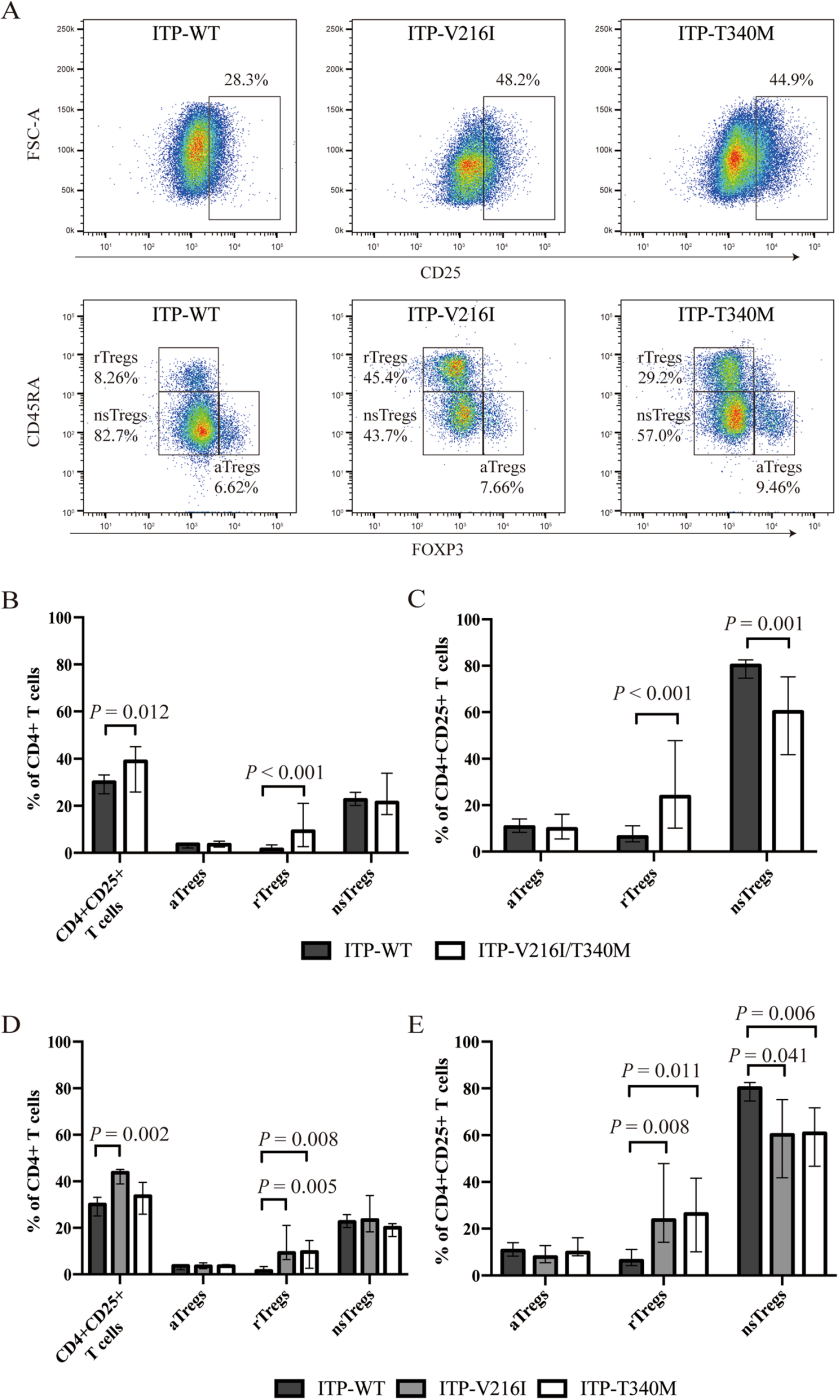
Percentage of Tregs subtypes with or without *TGFBR2* mutations. Tregs were defined as CD4^+^CD25^hi^Foxp3^+^ cells. Treg compartments, namely aTreg, rTreg, and nsTreg, were further defined as Foxp3^hi^CD45RA^−^, Foxp3^int^CD45RA^+^, and Foxp3^int^CD45RA^−^ cells, respectively among Tregs. **A** Representative flow plot of CD4^+^CD25^+^ T cells and Tregs subsets. **B** Percentage of CD4^+^CD25^+^ T cells and Tregs subsets in CD4^+^ T cells in ITP patients with and without *TGFBR2* mutations. **C** Percentage of Tregs subsets in CD4^+^CD25^+^ T cells in ITP patients with and without *TGFBR2* mutations. **D** Percentage of CD4^+^CD25^+^ T cells and Tregs subsets in CD4^+^ T cells among the ITP patients with V216I mutation, with T340M mutation and wild type. **E** Percentage of Tregs subsets in CD4^+^CD25^+^ T cells among ITP patients with V216I mutation, with T340M mutation and wild type

Based on the expression of CD45RA and Foxp3, human Tregs can be categorized into 3 phenotypically and functionally distinct subsets: aTregs, rTregs and n-s Tregs. Representative flow plots of CD4^+^CD25^+^ T cells and Tregs subsets of ITP patients with V216I mutation, T340M mutation and WT were shown in Fig. 1A. The frequency of total CD4^+^CD25^+^ T cells and rTregs in the CD4^+^ T cells was significantly elevated in ITP-V216I/T340M group (*p* = 0.012 and *p* < 0.001, respectively, Fig. 1B). The ITP-V216I/T340M group possessed significant higher rTregs and decreased n-s Tregs percentage than the ITP-WT group did (*p* < 0.001 and *p* = 0.001, Fig. 1C). The percentage of CD4^+^CD25^+^ T cells in CD4^+^ T cells was elevated in the V216I group (*p* = 0.002, Fig. 1D). Both the ITP-V216I group and ITP-T340M group all possessed a higher level of rTregs compared with ITP-WT group (*p* = 0.005, *p* = 0.008, respectively, Fig. 1D). ITP-V216I group and ITP-T340M group had higher rTregs percentage and decreased n-s Tregs percentage (Fig. 1E).

### The impact of TGFBR2 variants in elderly primary ITP patients

Among 44 elderly primary ITP patients, *TGFBR2* variants were found in 6 patients, including two V216I and four T340M heterozygote carriers. Clinical characteristics were comparable between elderly primary ITP patients with and without *TGFBR2* variants, although heterozygote carriers demonstrated an elevated rate to achieve PR (83.3% vs. 31.6%, *p* = 0.016) after first-line regimens (Table 3).

**Table 3:** Comparison between elderly primary ITP patients with and without *TGFBR2* variants

	Elderly ITP (n = 44)	Elderly ITP with *TGFBR2* variants (n = 6)	Elderly ITP without *TGFBR2* variants (n = 38)	*p* value
Female sex, n (%)	26 (59.1)	5 (83.3)	21 (55.3)	0.194
Age, years	65 ± 10	61 ± 13	66 ± 9	0.233
Platelet count, × 10^9^/L	16 ± 8	13 ± 10	17 ± 16	0.615
Response, n (%)				
CR	18 (40.9)	1 (16.7)	17 (44.7)	0.194
PR	17 (38.6)	5 (83.3)	12 (31.6)	0.016
NR	9 (20.5)	0	9 (23.7)	0.181
Positive ANA, n (%)	12 (27.3)	1 (16.7)	11 (28.9)	0.530
Lymphocyte subgroups, %				
CD19^+^	16 ± 10	14 ± 13	17 ± 10	0.659
CD3^+^CD4^+^	37 ± 11	39 ± 12	37 ± 11	0.765
CD3^+^CD8^+^	27 ± 14	21 ± 9	29 ± 14	0.366
CD16^+^CD56^+^	14 ± 7	23 ± 13	12 ± 6	0.294
Tregs, %				
CD4^+^CD25^hi^Foxp3^+^	1.2 ± 0.9	1.4 ± 0.8	1.1 ± 0.9	0.594
CD4^+^CD25^hi^Foxp3^+^IFN-γ^+^	8.3 ± 4.8	6.1 ± 4.2	9.0 ± 4.9	0.261
CD4^+^CD25^hi^Foxp3^+^IL-13^+^	7.4 ± 4.5	5.7 ± 4.1	7.9 ± 4.6	0.349
CD4^+^CD25^hi^Foxp3^+^IL-17^+^	16.9 ± 13.8	16.8 ± 8.8	17.0 ± 14.7	0.985
CD4^+^CD25^hi^Foxp3^hi^CD45RA^−^	21.8 ± 16.3	26.2 ± 3.2	20.5 ± 18.5	0.466
CD4^+^CD25^hi^Foxp3^int^CD45RA^+^	21.4 ± 12.3	23.1 ± 10.2	20.9 ± 13.1	0.702
CD4^+^CD25^hi^Foxp3^int^CD45RA^−^	51.3 ± 15.0	46.5 ± 7.1	52.9 ± 16.6	0.371

CR: complete remission; PR: partial remission; NR: no remission; ANA: anti-nuclear antibody; Treg: regulatory T cell

**Fig. 2:**
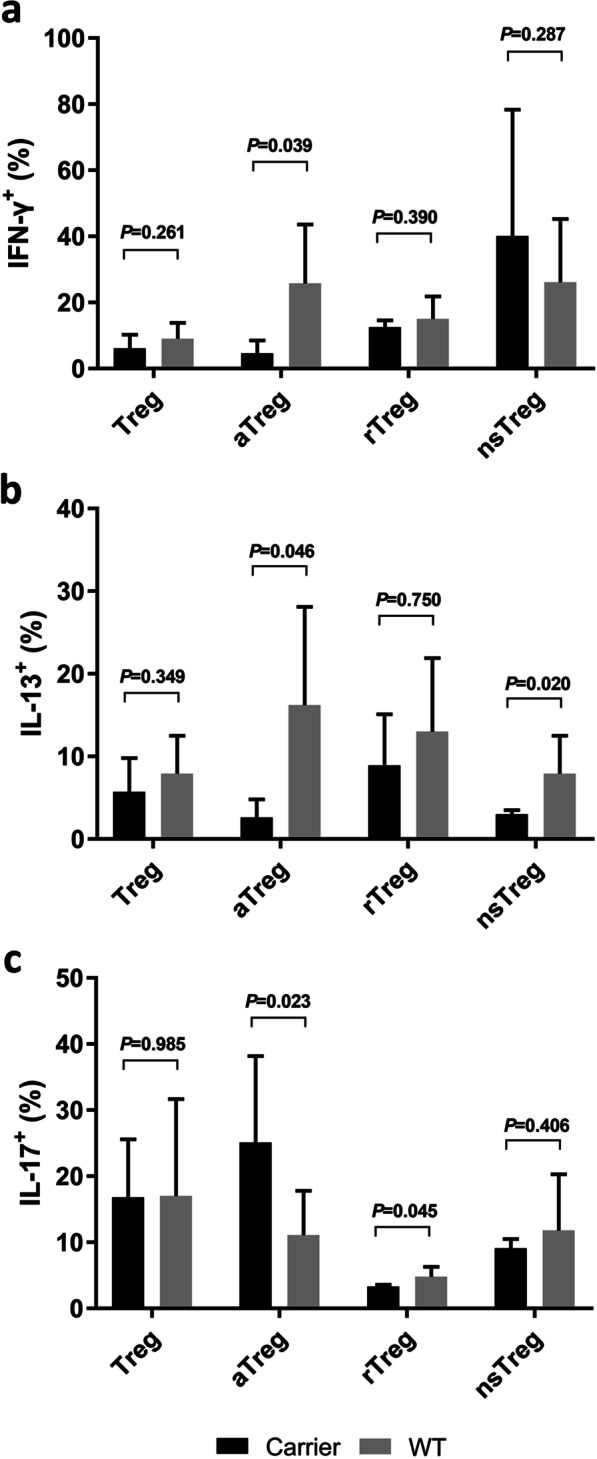
Co-expression of IFN-γ (**A**), IL-13 (**B**), and IL-17 (**C**) among Tregs and Treg compartments between elderly primary ITP patients with and without *TGFBR2* variants. **A**–**C** Percentage of Tregs, aTregs, rTregs and n-sTregs that produce IL-17, IL-13 and IFN-γ in ITP patients with and without *TGFBR2* mutations

Lymphocyte subgroups, total Tregs, Treg compartments, and Treg plasticity in total Tregs were all comparable between elderly primary ITP patients with and without *TGFBR2* variants. However, *TGFBR2* variant carriers demonstrated markedly elevated co-expression of IL-17 [(25.1 ± 13.1)% vs. (11.1 ± 6.7)%, *p* = 0.023] and decreased co-expression of both IFN-γ [(4.6 ± 3.9)% vs. (25.8 ± 17.8)%, *p* = 0.039] and IL-13 [(2.6 ± 2.2)% vs. (16.2 ± 11.9)%, *p* = 0.046] in the aTreg compartment, as well as decrease co-expression of IL-17 [(3.3 ± 0.3)% vs. (4.8 ± 1.5)%, *p* = 0.045] in the rTreg and IL-13 [(3.0 ± 0.5)% vs. (7.9 ± 4.6)%, *p* = 0.020] in the nsTreg compartment (Fig. 2).

## Discussion

The present study was among the first to demonstrate additional aberrations in proinflammatory plasticity of Tregs and aTregs among elderly primary ITP patients. The principal findings from our study were three-fold. First, primary ITP patients in general displayed proinflammatory plasticity of Tregs towards Th17 paradigm. Second, elderly primary ITP patients displayed further skewed Treg plasticity towards Th17 paradigm and elevated incidence of *TGFBR2* variants than younger patients. Third, elderly primary ITP patients with *TGFBR2* variants displayed skewed Treg plasticity towards Th17 paradigm in aTreg instead of rTreg or nsTreg compartments than those without *TGFBR2* variants. Our findings highlighted the unique immune status of elderly primary ITP patients, and advocated the establishment of pertinent treatment strategies for these patients.

The proinflammatory plasticity of Tregs has been described in various autoimmune disorders. It has been established that these biphenotypic or exFoxp3 Tregs could adopt effector Th phenotype in a reversible way [21, 22]. The exact biological function of biphenotypic Tregs is yet to be concluded. Based on the observations that biphenotypic Tregs always adopt the same type of effector Th markers as the predominant Th functional group in autoimmune diseases, it is highly probable that the skewed proinflammatory plasticity indicates Treg dysfunction and incompetence to counter over-activated immune responses [23, 24]. The imbalance of Th17/Treg ratio in primary ITP patients has been well-addressed as the signature for its immune microenvironment [7, 9, 25, 26]. The present study confirmed the over-expression of IL-17 instead of IFN-γ or IL-13 in Tregs among primary ITP patients, which advocated the hypothesis that Th17 participated in the pathogenesis of primary ITP as the predominant Th functional group.

The most intriguing finding from the present study was that elderly primary ITP patients seemed to display aggravated Treg dysfunction in the form of elevated co-expression of IL-17. It should be noted that an onset age of 50 years was applied as a dichotomous approach to categorize our cohort of adult primary ITP patients. Primary ITP in this elderly population demonstrated a relatively equal sex distribution as previously reported, along with skewed Treg plasticity implying aberrant immune modulation. Senescent Tregs, either from elderly individuals or after repeatedly inflammatory stimulation, have been shown to frequently lose Foxp3 expression and to reprogram their activity towards effector Th paradigm [27, 28]. Our observation might be among the early evidences indicating the role of Treg senescence in the pathogenesis of primary ITP, although further investigations are required to illustrate the association between T cell senescence markers and the extent of Treg dysfunction.

TGF-β signaling is crucial in the development and differentiation of Tregs [29]. Canonical TGF-β signaling pathway comprises of TGF-β I/II receptors and Smad proteins to be phosphylated and translocated into nucleus to regulate the transcription of target genes [30]. Rare germline *TGFBR1*/*2* mutations lead to Loeys-Dietz syndrome (LDS) which is a sinister genetic connective tissue disorder characterized by early-onset aortic aneurysm, craniofacial features, and allergic diseases [31]. Frischmeyer-Guerrerio et al*.* [17] described elevated co-expression of IL-13 in Tregs from a cohort of LDS patients with eosinophilic gastrointestinal disease. On the other hand, common *TGFBR2* variants, especially V216I and T340M in the East Asian population, have been reported to be potential biomarkers in aortic aneurysmal diseases [32] and pancreatic neoplasms [33]. It should be noted that the incidence of *TGFBR2* variants was not elevated in primary ITP patients compared to that of the general East Asian population. Therefore, it is unlikely that common *TGFBR2* variants lead to the pathogenesis of primary ITP. However, the present study revealed an elevated incidence of 13.6% of two common *TGFBR2* variants in elderly primary ITP patients who displayed senescence related Treg dysfunction, indicating potential genetic susceptibility to develop autoimmune diseases in their middle to late adulthood instead of earlier ages, as well as impaired immune tolerance to benefit from corticosteroids.

Further analysis revealed that the carrier status of *TGFBR2* variants predominantly affected the proinflammatory plasticity of aTreg compartment instead of Treg as a whole. Based on the expression of surface CD45RA and intracellular Foxp3, Tregs are categorized into aTreg, rTreg, and nsTreg compartments. As terminally differentiated cells, aTregs suppress effector Th function and immune responses, while rTregs are capable of proliferation and conversion to aTregs upon stimulation and nsTregs production of proinflammatory cytokines [20, 34, 35]. We have found in previous investigations [36] that higher turnover rate from rTregs to aTregs could be associated with superior response to first-line corticosteroids among newly diagnosed primary ITP patients. Therefore, it was plausible that the impaired aTreg function might be one of the underlying causes for elevated rate to achieve PR among elderly primary ITP patients with *TGFBR2* V216I and T340M variants.

With the progress of treatment options for primary ITP, the first-line regimens for elderly primary ITP patients might require further evaluation. Considering the potential adverse reactions associated with glucocorticoids, early application of thrombopoietin receptor agonists is appealing [37, 38]. In additional to the existing factors that affect glucocorticoid response, the specificity of platelet autoantibodies in particular [39, 40], Treg senescence could be a promising perspective to provide novel evidence for treatment options. Since the incidence of primary ITP increases with aging, more attention should be paid to the elderly patient population among whom practical parameters for the evaluation of Treg dysfunction might be of great clinical value.

The present study should be viewed in the light of its limitations. The retrospective nature and small cohort size limited the interpretation power of its findings. The molecule mechanism of how Treg differentiation and senescence are affected by *TGFBR2* variants is still unclear. Further investigations are warranted to establish the rationale between Treg senescence and Treg plasticity in larger cohorts of elderly primary ITP patients. Comprehensive illustration of immune aberrations in elderly primary ITP patients could be of great importance to update clinical recommendations for early application of thrombopoietin receptor agonists and tapering of corticosteroids.

## Conclusions

Primary ITP demonstrated skewed Treg plasticity towards Th17 paradigm, which was further aggravated in elderly patients presenting ITP since their 6th decade of age. Heterozygous carriers of *TGFBR2* variants were more prevalent in elderly patients, and affected the proinflammatory plasticity of aTreg compartment. Evaluation of Treg dysfunction and senescence could benefit the optimization of management for elderly primary ITP patients.

## Data Availability

All data generated or analyzed during this study are included in this published article. The datasets used or analyzed during the current study are available from the corresponding author on reasonable request.

## References

[1] Schoonen WM, Kucera G, Coalson J, Li L, Rutstein M, Mowat F, Fryzek J, Kaye JA (2009). Epidemiology of immune thrombocytopenic purpura in the General Practice Research Database. Br J Haematol.

[2] Cooper N, Ghanima W (2019). Immune thrombocytopenia. N Engl J Med.

[3] Michel M, Rauzy OB, Thoraval FR, Languille L, Khellaf M, Bierling P, Godeau B (2011). Characteristics and outcome of immune thrombocytopenia in elderly: results from a single center case-controlled study. Am J Hematol.

[4] Zhou H, Fu R, Wang H, Zhou F, Li H, Zhou Z, Zhang L, Yang R (2013). Immune thrombocytopenia in the elderly: clinical course in 525 patients from a single center in China. Ann Hematol.

[5] Ray D, Yung R (2018). Immune senescence, epigenetics and autoimmunity. Clin Immunol.

[6] Cines DB, Blanchette VS (2002). Immune thrombocytopenic purpura. N Engl J Med.

[7] Ji L, Zhan Y, Hua F, Li F, Zou S, Wang W, Song D, Min Z, Chen H, Cheng Y (2012). The ratio of Treg/Th17 cells correlates with the disease activity of primary immune thrombocytopenia. PLoS ONE.

[8] Lu Y, Cheng L, Li F, Ji L, Shao X, Wu B, Zhan Y, Liu C, Min Z, Ke Y (2019). The abnormal function of CD39(+) regulatory T cells could be corrected by high-dose dexamethasone in patients with primary immune thrombocytopenia. Ann Hematol.

[9] Semple JW, Rebetz J, Maouia A, Kapur R (2020). An update on the pathophysiology of immune thrombocytopenia. Curr Opin Hematol.

[10] Gratz IK, Campbell DJ (2014). Organ-specific and memory treg cells: specificity, development, function, and maintenance. Front Immunol.

[11] Weist BM, Kurd N, Boussier J, Chan SW, Robey EA (2015). Thymic regulatory T cell niche size is dictated by limiting IL-2 from antigen-bearing dendritic cells and feedback competition. Nat Immunol.

[12] Oestreich KJ, Weinmann AS (2012). Master regulators or lineage-specifying? Changing views on CD4+ T cell transcription factors. Nat Rev Immunol.

[13] Sakaguchi S, Yamaguchi T, Nomura T, Ono M (2008). Regulatory T cells and immune tolerance. Cell.

[14] Dominguez-Villar M, Hafler DA (2018). Regulatory T cells in autoimmune disease. Nat Immunol.

[15] Scheinecker C, Göschl L, Bonelli M (2020). Treg cells in health and autoimmune diseases: new insights from single cell analysis. J Autoimmun.

[16] Barbi J, Pardoll D, Pan F (2014). Treg functional stability and its responsiveness to the microenvironment. Immunol Rev.

[17] Frischmeyer-Guerrerio PA, Guerrerio AL, Oswald G, Chichester K, Myers L, Halushka MK, Oliva-Hemker M, Wood RA, Dietz HC (2013). TGFbeta receptor mutations impose a strong predisposition for human allergic disease. Sci Transl Med.

[18] Rodeghiero F, Stasi R, Gernsheimer T, Michel M, Provan D, Arnold DM, Bussel JB, Cines DB, Chong BH, Cooper N (2009). Standardization of terminology, definitions and outcome criteria in immune thrombocytopenic purpura of adults and children: report from an international working group. Blood.

[19] Liang Y, Rascati K, Richards K (2021). Prevalence of primary immune thrombocytopenia and related healthcare resource utilization among Texas Medicaid beneficiaries. Curr Med Res Opin.

[20] Miyara M, Yoshioka Y, Kitoh A, Shima T, Wing K, Niwa A, Parizot C, Taflin C, Heike T, Valeyre D (2009). Functional delineation and differentiation dynamics of human CD4+ T cells expressing the FoxP3 transcription factor. Immunity.

[21] Komatsu N, Okamoto K, Sawa S, Nakashima T, Oh-hora M, Kodama T, Tanaka S, Bluestone JA, Takayanagi H (2014). Pathogenic conversion of Foxp3+ T cells into TH17 cells in autoimmune arthritis. Nat Med.

[22] Butcher MJ, Filipowicz AR, Waseem TC, McGary CM, Crow KJ, Magilnick N, Boldin M, Lundberg PS, Galkina EV (2016). Atherosclerosis-driven Treg plasticity results in formation of a dysfunctional subset of plastic IFNγ+ Th1/Tregs. Circ Res.

[23] Koenen HJ, Smeets RL, Vink PM, van Rijssen E, Boots AM, Joosten I (2008). Human CD25highFoxp3pos regulatory T cells differentiate into IL-17-producing cells. Blood.

[24] Beriou G, Costantino CM, Ashley CW, Yang L, Kuchroo VK, Baecher-Allan C, Hafler DA (2009). IL-17-producing human peripheral regulatory T cells retain suppressive function. Blood.

[25] Lyu M, Li Y, Hao Y, Lyu C, Huang Y, Sun B, Li H, Xue F, Liu X, Yang R (2019). CCR6 defines a subset of activated memory T cells of Th17 potential in immune thrombocytopenia. Clin Exp Immunol.

[26] Li J, Hua M, Hu X, Zhang Y, Feng Q, Qiu J, Shao L, Li N, Hou M, Peng J (2020). Dexamethasone suppresses the Th17/1 cell polarization in the CD4(+) T cells from patients with primary immune thrombocytopenia. Thromb Res.

[27] Kornete M, Mason E, Istomine R, Piccirillo CA (2017). KLRG1 expression identifies short-lived Foxp3(+) T(reg) effector cells with functional plasticity in islets of NOD mice. Autoimmunity.

[28] González-Osuna L, Sierra-Cristancho A, Rojas C, Cafferata EA, Melgar-Rodríguez S, Cárdenas AM, Vernal R (2021). Premature senescence of T-cells favors bone loss during osteolytic diseases. A new concern in the osteoimmunology arena. Aging Dis.

[29] Zhu J, Paul WE (2008). CD4 T cells: fates, functions, and faults. Blood.

[30] Gu AD, Wang Y, Lin L, Zhang SS, Wan YY (2012). Requirements of transcription factor Smad-dependent and -independent TGF-β signaling to control discrete T-cell functions. Proc Natl Acad Sci USA.

[31] Loeys BL, Schwarze U, Holm T, Callewaert BL, Thomas GH, Pannu H, De Backer JF, Oswald GL, Symoens S, Manouvrier S (2006). Aneurysm syndromes caused by mutations in the TGF-beta receptor. N Engl J Med.

[32] Wu B, Li J, Wang Y, Cheng Y, Wang C, Shu X (2021). Recurrent germline mutations as genetic markers for aortic root dilatation in bicuspid aortic valve patients. Heart Vessels.

[33] Javle M, Li Y, Tan D, Dong X, Chang P, Kar S, Li D (2014). Biomarkers of TGF-β signaling pathway and prognosis of pancreatic cancer. PLoS ONE.

[34] Shi G, Han J, Liu G, Hao Y, Ma Y, Li T, Wu X, Zhang H, Liu Y, Wang B (2014). Expansion of activated regulatory T cells by myeloid-specific chemokines via an alternative pathway in CSF of bacterial meningitis patients. Eur J Immunol.

[35] Luo CT, Liao W, Dadi S, Toure A, Li MO (2016). Graded Foxo1 activity in Treg cells differentiates tumour immunity from spontaneous autoimmunity. Nature.

[36] Cheng L, Liu C, Li F, Wu B, Min Z, Chen P, Zhan Y, Ke Y, Hua F, Yuan L (2019). The prediction value of Treg cell subtype alterations for glucocorticoid treatment in newly diagnosed primary immune thrombocytopenia patients. Thromb Res.

[37] Dou X, Yang R (2019). Current and emerging treatments for immune thrombocytopenia. Expert Rev Hematol.

[38] Ghanima W, Cooper N, Rodeghiero F, Godeau B, Bussel JB (2019). Thrombopoietin receptor agonists: ten years later. Haematologica.

[39] Zeng Q, Zhu L, Tao L, Bao J, Yang M, Simpson EK, Li C, van der Wal DE, Chen P, Spring CM (2012). Relative efficacy of steroid therapy in immune thrombocytopenia mediated by anti-platelet GPIIbIIIa versus GPIbα antibodies. Am J Hematol.

[40] Li J, van der Wal DE, Zhu G, Xu M, Yougbare I, Ma L, Vadasz B, Carrim N, Grozovsky R, Ruan M (2015). Desialylation is a mechanism of Fc-independent platelet clearance and a therapeutic target in immune thrombocytopenia. Nat Commun.

